# Editorial Expression of Concern on article ‘Functional dissection of the C-terminal domain of type II DNA topoisomerase from the kinetoplastid hemoflagellate *Leishmania donovani*’

**DOI:** 10.1093/nar/gkab171

**Published:** 2021-03-08

On 15 September 2003, NAR published the article ‘Functional dissection of the C-terminal domain of type II DNA topoisomerase from the kinetoplastid hemoflagellate *Leishmania donovani*’ by Tanushri Sengupta, Mandira Mukherjee, Chhabinath Mandal, Aditi Das, and Hemanta K. Majumder (1).

The Editors were recently alerted by a reader that the bacterial colonies depicted in Figure 2B have been duplicated within and across the figure panels despite different using different clones and incubation conditions.

In Figure 3C, multiple lanes have also clearly been spliced in.

Details of the duplications and splicing have been published in PubPeer: https://pubpeer.com/publications/34E9446142CBE8FCD55311F1EB9C1C

The Authors are unable to provide the original data or a reasonable explanation for the image manipulations. Pending the results of a full investigation at the Authors’ institution, the Editors advise Readers to examine the details of this study with care.

Keith R. Fox, Barry L. Stoddard

Senior Executive Editors
